# Fungal Endophytes of *Alpinia officinarum* Rhizomes: Insights on Diversity and Variation across Growth Years, Growth Sites, and the Inner Active Chemical Concentration

**DOI:** 10.1371/journal.pone.0115289

**Published:** 2014-12-23

**Authors:** Li Shubin, Huang Juan, Zhou RenChao, Xu ShiRu, Jin YuanXiao

**Affiliations:** Guangdong Provincial Key Lab of Biotechnology for Plant Development, College of Life Sciences, South China Normal University, Guangzhou, China; USDA Forest Service - RMRS, United States of America

## Abstract

In the present study, the terminal-restriction fragment length polymorphism (T-RFLP) technique, combined with the use of a clone library, was applied to assess the baseline diversity of fungal endophyte communities associated with rhizomes of *Alpinia officinarum* Hance, a medicinal plant with a long history of use. A total of 46 distinct T-RFLP fragment peaks were detected using *Hha*I or *Msp*I mono-digestion-targeted, amplified fungal rDNA ITS sequences from *A. officinarum* rhizomes. Cloning and sequencing of representative sequences resulted in the detection of members of 10 fungal genera: *Pestalotiopsis*, *Sebacina*, *Penicillium*, *Marasmius*, *Fusarium*, *Exserohilum*, *Mycoleptodiscus*, *Colletotrichum*, *Meyerozyma*, and *Scopulariopsi*s. The T-RFLP profiles revealed an influence of growth year of the host plant on fungal endophyte communities in rhizomes of this plant species; whereas, the geographic location where *A. officinarum* was grown contributed to only limited variation in the fungal endophyte communities of the host tissue. Furthermore, non-metric multidimensional scaling (NMDS) analysis across all of the rhizome samples showed that the fungal endophyte community assemblages in the rhizome samples could be grouped according to the presence of two types of active indicator chemicals: total volatile oils and galangin. Our present results, for the first time, address a diverse fungal endophyte community is able to internally colonize the rhizome tissue of *A. officinarum*. The diversity of the fungal endophytes found in the *A. officinarum* rhizome appeared to be closely correlated with the accumulation of active chemicals in the host plant tissue. The present study also provides the first systematic overview of the fungal endophyte communities in plant rhizome tissue using a culture-independent method.

## Introduction

Fungal endophytes, which colonize plants internally without apparent adverse effects, have been found in almost all plant species examined to date [1]. Some fungal endophytes are mutualistic, conferring tolerance to biotic and abiotic stress on their hosts and therefore enhancing the survival and growth of plants in adverse conditions [1], [2], [3], [4]. Fungal endophytes can sometimes be an alternative source of important plant secondary metabolites because many fungal endophytes show the ability to produce the same desirable natural products that are produced by their respective host plants, while also producing novel active chemicals, including potential anti-cancer, anti-fungal, anti-diabetic and immunosuppressant compounds [1,5 6,7].

The diversity and population composition of fungal endophytes of plants are highly variable. The variation of these fungal communities under different conditions, such as different host plant species and genotypes, host developmental stages, host tissue types, growth locations and growth seasons, have been intensely investigated [8], [9], [10], [11], [12], [13], [14], [15], [16], [17]. However, the specific mechanisms driving variations in fungal endophyte communities as influenced by abiotic and/or biotic factors are poorly understood.

Host species and organ types are major factors shaping fungal endophyte communities. Rhizomes, or underground plant stems, are of fundamental importance for plant competitiveness and growth [18]. In addition, many rhizome-derived or rhizome-stored compounds are medicinally or economically important [18], [19]. As a result of adaptation to different physiological conditions in plants, rhizome tissues may be colonized by a characteristic fungal community. Thus, increasing our understanding of fungal endophyte communities within rhizomes could significantly impact our understanding of how important medicinal compounds are produced, how plants are able to survive severe drought and cold stress, and how to control important weed species. However, previous reports documenting the study of rhizome fungal endophytes are quite limited. Furthermore, the majority of current information about fungal endophytes of rhizomes has been obtained using culture-dependent methods, mainly focused on the isolation of active substance-producing strains [20], [21], [22], [23]. No studies have addressed the influence of either host plant traits or environmental conditions on fungal endophyte communities of rhizomes.

*Alpinia officinarum* Hance (lesser galanga, Zingiberaceae) is a perennial medicinal plant that is mainly distributed in the tropical and subtropical regions of Southeast Asia. The most medicinally active part of the plant is the rhizome, which is characterized by dark, reddish brown coloring and a strong aromatic odor. The rhizomes of *A. officinarum* are widely used in China, India, and other Asian countries for relieving stomach aches, treating colds, invigorating the circulatory system, and reducing swelling [24], [25]. This plant species has also been used in Europe as a spice for over 1,000 years [25]. However, no available research reports are available on the fungal endophytes of this medicinal plant species. Furthermore, endophyte studies are also quite rare for any plant species within the Zingiberaceae family [21], [23].

Culture-dependent isolation methods are important for the study of fungal endophytes, especially when isolating bioactive compound-producing endophytes. However, culture-dependent methods for quantifying multiple microbial communities are limited by critical factors, such as short-duration sampling times and the inability to survey/identify slow-growing, non-culturable, or nonviable microbes. Consequently, the quantitative diversity of the fungal endophyte communities is often underestimated [12], [26]. In recent years, studies on endophyte communities have often employed culture-independent methods, such as denaturing gradient gel electrophoresis (DGGE), high-throughput, next-generation sequencing (454 pyrosequencing), and terminal-restriction fragment length polymorphism (T-RFLP) techniques [27], [28], [29], [30], [31], [32]. Among these techniques, conventional T-RFLP has been extensively applied for comparison of multiple microbial communities because it can provide distinct profiles that reflect not only the taxonomic composition of the sampled community but also the relative abundance of individual species in the sampled community at a lower cost and more rapidly than other methods [32], [33], [34]. In the present study, T-RFLP analysis, combined with the use of a clone library, was applied to assess the fungal endophyte communities of *A. officinarum*. Because little is known about the fungal endophyte communities of *A. officinarum* and of plant rhizome tissue in general, the goals of this study were to 1) provide baseline information about the fungal endophyte communities of *A. officinarum* rhizomes; 2) examine potential influence of factors, such as the growth age and growth site of the host plant, on diversity of the fungal endophyte community in the rhizome; and 3) assess potential links among fungal endophyte assemblages and active host chemicals in *A. officinarum* rhizomes and determine relationships among the active chemical content of the host plant, fungal endophyte community assemblages, and changing host-plant conditions.

## Materials and Methods

### *A. officinarum* rhizome samples

Rhizome samples from *A. officinarum* plants grown at nine different sites were collected from June to July of 2011. No specific permissions for any locations/activities involved in the present study were required and the study area did not involve endangered or protected species. Rhizome samples LT-1-RZ, LT-2-RZ, LT-3-RZ, and LT-4-RZ were obtained from 1- to 4-year-old *A. officinarum* plants planted at site LT (20.3350, 110.3122). Rhizome samples HA-RZ, HN-RZ, GZ-RZ, GX-RZ, GY-RZ, FJ-RZ, KM-RZ, and QJ-RZ were collected from approximately 3- to 4-year-old *A. officinarum* plants growing naturally at sites HA (20.2831, 110.2089), HN (18.7350, 110.2328), GZ (23.1800, 113.3647), GX (22.5311, 108.3900), GY (22.8792, 112.3894), FJ (24.7206, 118.4750), KM (25.1433, 102.7417), and QJ (20.4528, 110.3619), respectively. The sampling sites were distributed across five different provinces of China: Guangdong, Guangxi, Hainan, Yuannan, and Fujian. Sites QJ, HA, and LT were geographically close, separated by less than 50 km, whereas the other sites were separated by >150 km (ranging from 150 to 1900 km) (S1 Fig.). For each site/year, at least five intact rhizomes were collected from different plants of the single species. For the analysis of the endophyte communities, each rhizome from a plant was placed separately into a sterile bag containing dry silica gel and stored at −20°C. Total DNA was extracted from the materials within 2 weeks after collection. For analysis of active chemicals, the collected rhizomes were placed separately into preservation bags, and the analysis was performed within 1 week after collection.

### Detection of active chemicals

The contents of two types of active indicator chemicals, total volatile oils and galangin, were determined for each *A. officinarum* rhizome. The collected rhizomes were washed under running tap water to remove dirt and other external debris. After drying to constant weight in an oven at 45°C, rhizomes of approximately 40 mm in length were finely ground. Galangin was detected by HPLC in methanol extracts of precisely weighed rhizome samples, according to the method described by Tao et al. [35]. The galangin content was calculated based on the calibration curve for a galangin standard (y = 10.264x+70.082, R^2^ = 0.9994) prepared under the same conditions and was expressed as mg per g of dried sample. The volatile oils were extracted via hydrodistillation for 4 h according to Appendix X D of the *Chinese Pharmacopoeia* [36], then collected and precisely weighed. The volatile oil content was expressed as mg per 100 g of dried sample. Three replicates were conducted for each year or site.

### Surface sterilization and total DNA extraction

The collected rhizomes were washed and then surface disinfested using the following immersion sequence: 75% ethanol for 1 min, NaOCl (6% available chlorine) for 5 min, and rinsing with sterile water three times. Following surface disinfestation, the outer layers of the rhizome were entirely removed under sterile conditions. Approximately 0.5 g of central part of the rhizome was subjected to total DNA extraction via the CTAB protocol using a commercial Genomic DNA Mini Preparation Kit with a Spin Column (Biotime Institute of Biotechnology, Shanghai, China), according to the manufacturer's protocol. The extracted DNA was resuspended in 50 µl of Tris-EDTA containing RNase (0.1 mg/ml) and stored at −70°C until analysis. The quality and quantity of the extracted DNA were assessed spectrophotometrically by calculating the absorbance at wavelengths of 260 nm (A_260_), 230 nm (A_230_), and 280 nm (A_280_), which was used to determine the A_260_/A_230_ and A_260_/A_280_ ratios.

### T-RFLP analysis

The fungal nuclear ribosomal DNA internal transcribed spacers 1 and 2 (rDNA ITS) containing the 5.8 s gene were amplified from the extracted DNA via PCR utilizing the 6-carboxyfluorescein (FAM)-labeled forward primer (5′-FAM- CTTGGTCATTTAGAGGAAGTAA-3′) [29] and the unlabeled reverse primer ITS4 (5′-TCCTCCGCTTATTGATATGC-3′). Each 25-µl PCR mixture contained 1 µl DNA extract (∼100 ng of DNA), 1 µl (l0 µM) each primer, 12.5 µl HotStart Taq PCR Master-Mix (TIANGEN, China), and 9.5 µl ddH_2_O. PCR was performed in a thermocycler (PTC-200, Bio-Rad Laboratories, Inc., USA) under the following conditions: 95°C (5 min), followed by 30 cycles at 94°C (30 s), 51.6°C (45 s), and 72°C (40 s), with a final extension step at 72°C (7 min). The resulting PCR products (ca. 600 bp) were eluted from the gels and then purified using a PCR Purification Kit (TaKaRa, Dalian, China). The products of three replicate PCR runs using the same template were pooled for subsequent restriction enzyme digestion. Restriction enzyme digestion was carried out in a total volume of 10 µl, containing 100 ng purified PCR product and 10 U *Msp*I or *Hha*I (Takara, Dalian, China) at 37°C for 3 h, followed by an inactivation step for 20 min at 65°C.

A 2-µl aliquot of the digested PCR product was mixed with 0.75 µl DNA size standard GeneScan TM-500 LIZR (Applied Biosystems, USA) and 7.25 µl Hi-Di Formamide (Applied Biosystems, USA), and the DNA fragments were then scanned in an ABI 3730 DNA Analyzer (Applied Biosystems, USA). Terminal restriction fragments peaks (T-RFs) with lengths between 50 and 550 bp and heights of ≥50 fluorescence units were included in the analysis [34]. The relative abundance of the T-RFs detected in each sample was expressed as the normalized peak height (the peak height divided by the cumulative peak height for a given sample) [34]. For each year or site, the DNA extraction followed by T-RFLP analysis was repeated in three independent rhizome samples derived from three different plants from a given year or site.

### Clone library construction, and taxonomic affiliation of T-RFs

The DNA extracts from each rhizome from the *A. officinarum* plants were diluted to the same final concentration (ca. 8 ng of DNA each) for use as template DNA. The rDNA ITS region was amplified from the DNA template with the unlabeled forward primer and ITS4 primer pair. The PCR reagents and conditions applied were the same as described above. The resulting amplicons were purified and subsequently ligated into the pGEM-T vector and transformed into *E. coli* cells (TOP 10 strain) using Invitrogen's TA-cloning kit (Invitrogen, Carlsbad, CA, USA), according to the manufacturer's protocol. The transformants were randomly selected through standard blue-white screening on X-Gal (3,4-cyclohexenoesculetin-β-D-galactopyranoside; Sigma-Aldrich Co.) plates, and subjected to amplification of the rDNA ITS region using the labeled forward primer and unlabeled ITS4 primer pair. The PCR products were confirmed via electrophoresis in a 2.0% agarose gel, then eluted from the gels and purified. All of the recovered sequences were subjected to mono-digestion T-RFLP analysis with both *Hha*I and *Msp*I, as described above. The cloned ITS sequences were further collapsed into distinct operational taxonomic units (OTUs), with each OTU defined as a distinct pattern of peaks in the *Hha*I and/or *Msp*I profiles. Representative sequences were determined in an ABI PRISM 3730 sequencer by the BGI-Shenzhen Corporation (Shenzhen, China). Sequences with potential chimeras were excluded from further analysis by using the CHIMERA_CHECK program of the Ribosomal Database Project (RDP) II database (http://fungene.cme.msu.edu/FunGenePipeline/chimera_check/form.spr). Sequence similarity searches were performed using the basic local alignment search tool (BLAST) and GenBank. The fungal taxa identifications were done based on DNA sequence identities/similarities based on GenBank BLASTs.

### Data analysis

The T-RFLP profiles generated for each sample were aligned to produce single datasets comprised of either the relative abundance or a value of 0 (an absence of a peak) for each T-RF detected. Three types of diversity indices were calculated from the datasets and applied to each community: Shannon's diversity index, Shannon's evenness index, and Berger-Parker's index [37]. Additionally, the pairwise similarities of the datasets from year-to-year, site-to-site, or group membership-to-group membership for the active chemical-based groups were also calculated from the datasets. Similarity measure matrices across all samples with two types of ecological community similarity indices, Jaccard's index and the Bray-Curtis similarity index, were represented using non-metric multidimensional scaling (NMDS) plots. These similarity analyses were performed using PAST Version 2.1 software at a 95% confidence level. Prior to similarity comparison and NMDS performance, the data from T-RF measurments of three independent rhizome samples derived from different plants of a given year/site were averaged and only the average values of the data were used to perform the analyses.

Significant differences among the rhizome samples concerning the chemical parameters and diversity indices were determined via one-way analysis of variance (ANOVA) with Duncan's multiple range test conducted at a confidence level >95% (p<0.05). These analyses were performed using SPSS Version 17.0 software.

## Results

### Differences in T-RFLP fingerprint profiles among years

**Figure 1:**
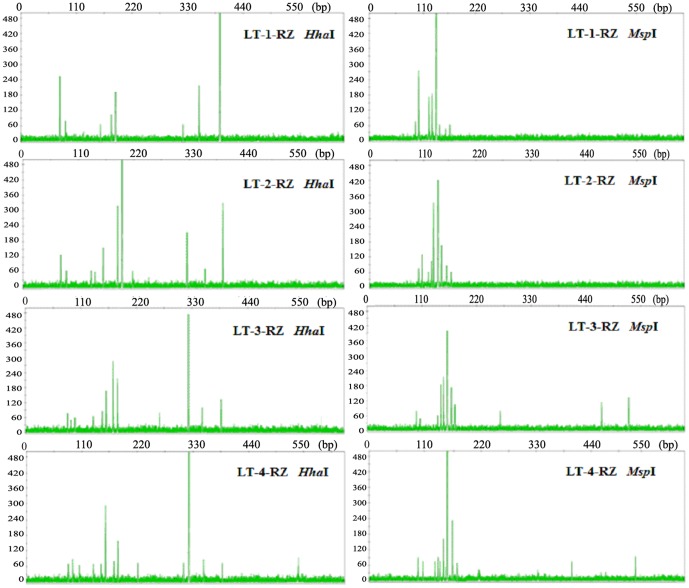
T-RFLP profiles for the rhizomes of *Alpinia officinarum* Hance plants at various ages. The profiles were produced via either *Hha*I or *Msp*I mono-digestion T-RFLP method targeting amplified fungal rDNA ITS sequences from the rhizomes of 1- to 4-year-old *A. officinarum* Hance planted at site LT. The T-RFLP analysis was repeated in three independent samples for each year, and the representative T-RFLP profiles are shown.

Fig. 1 shows the T-RFLP fingerprint profiles of targeted amplified fungal rDNA ITS sequences obtained from the rhizome samples of 1- to 4-year-old *A. officinarum* plants growing at the same site (LT). The number of total T-RFs from 1-, 2-, 3-, and 4-year-old rhizomes were determined to be 8, 11, 12, and 14, respectively, for *Hha*I cleavage, and 8, 9, 11, and 12, respectively, for *Msp*I cleavage (Fig. 1). For both the *Hha*I and *Msp*I profiles, there was a slight increase in the richness of T-RFs with increasing plant age. The major T-RFs (defined as the T-RFs displaying a relative abundance of more than 5% of the total abundance in their respective T-RFLP profiles) and the most dominant T-RFs for each sample were variably represented among the rhizomes. In particular, the profiles from the 4-year-old rhizome sample consisted of six major T-RFs (three from each cleavage), with a 325-bp T-RF obtained from *Hha*I cleavage and a 148-bp T-RF from *Msp*I cleavage showing strong dominance (accounting for more than 50% of the total T-RF peak heights in the two profiles for the sample). These 4-year-old, rhizome sample profiles were different from the profiles of the 1- to 3-year-old rhizome samples, in which other major T-RFs were detected, which either shared or replaced the robust dominance of the 325-bp *Hha*I T-RF and the 148-bp *Msp*I T-RF (Fig. 1). Based on the obtained T-RFLP profiles, three types of diversity indices were calculated, which were different depending on the age of the host plant (Table 1). For both the *Hha*I and *Msp*I T-RFLP profiles, Shannon's diversity index increased during the first 3 years of host plant growth and then decreased. The Shannon's evenness index was higher during years 2 and 3, whereas the Berger-Parker index was lower during years 2 and 3 (Table 1). The pairwise Bray-Curtis similarities from year to year were also calculated, showing a range of 0.32 to 0.65. The results indicated that the rhizome fungal endophyte communities changed with host plant age (growth year).

**Table 1:** T-RFLP profile-based diversity estimates for the rhizomes of *Alpinia officinarum* Hance plants at various ages.

	Shannon diversity index (*Hha*I)	Shannon diversity index (*Msp*I)	Shannon's evenness (*Hha*I)	Shannon's evenness (*Msp*I)	Berger-Parker's index (*Hha*I)	Berger-Parker's index (*Msp*I)
LT-4-RZ	1.78±0.06a	1.66±0.04d	0.42±0.02a	0.41±0.03a	0.54±0.01d	0.57±0.02d
LT-3-RZ	2.13±0.01c	2.21±0.02b	0.65±0.01c	0.70±0.02b	0.29±0.01b	0.31±0.02b
LT-2-RZ	1.94±0.07b	2.05±0.05c	0.76±0.01d	0.78±0.01c	0.31±0.01a	0.33±0.01b
LT-1-RZ	1.49±0.03a	1.74±0.03a	0.52±0.01b	0.64±0.02b	0.58±0.01c	0.38±0.00c

The values were calculated separately based on the data from measurements of three independent rhizome samples for each year and are expressed as the mean ± SD. The letters in each lane indicate significant differences (p<0.05).

### Differences and similarities between T-RFLP fingerprints profiles among sites

**Figure 2:**
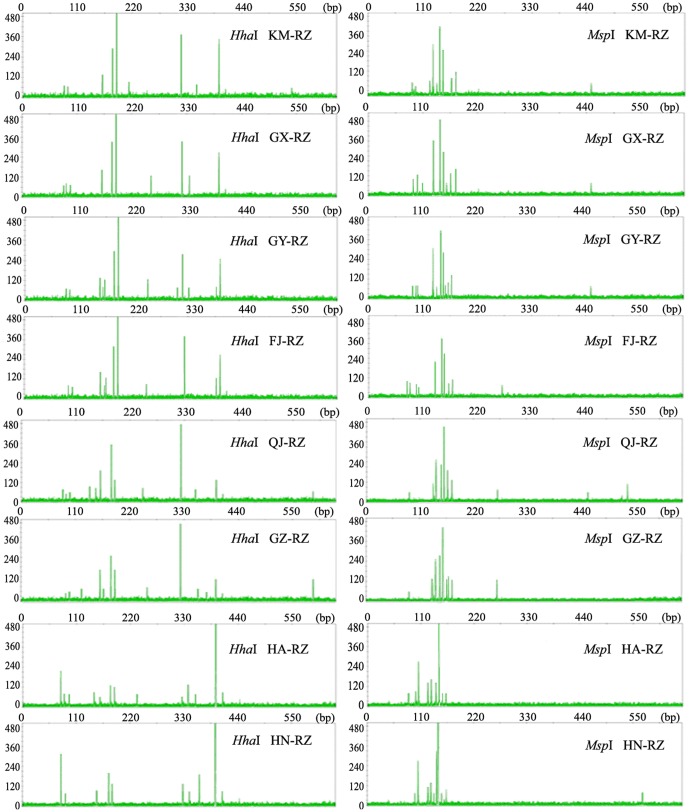
T-RFLP profiles for the rhizomes of *Alpinia officinarum* Hance plants from various growth sites. The profiles were generated via either *Hha*I or *Msp*I mono-digestion T-RFLP method targeting amplified fungal rDNA ITS sequences from the rhizomes of *A. officinarum* grown at eight different sites at varying geographic distances from each other. The T-RFLP analysis was repeated in three independent samples for each site, and representative T-RFLP profiles are shown.

Fig. 2 shows the T-RFLP fingerprint profiles detected from *A. officinarum* rhizomes from various growth sites, and the T-RFLP profile-based diversity estimates for the rhizomes are presented in Table 2. The number of total T-RFs for each site varied from 10 to 14 for the *Hha*I cleavage and from 8 to 13 for the *Msp*I cleavage. Only 11 of 46 T-RFs were shared among the samples from all eight sites, whereas the other T-RFs were variably present among sites (Fig. 2). Conversely, the T-RFLP profiles from the rhizomes collected from particular sites also displayed similarities. Rhizome samples FJ-RZ, GY-RZ, KM-RZ, and GX-RZ, obtained from sites FJ, GY, KM, and GX, shared the most dominant T-RF and most of the major T-RFs (Fig. 2). Furthermore, the diversity indices calculated from the T-RFLP profiles of these samples were all relatively close and showed no significant differences between the sites (p≥0.05) in most cases. The Bray-Curtis similarities between the sites were calculated to be 0.77–0.83, demonstrating similarity in T-RFLP profiles among different sites. The rhizome samples from sites HA and HN (HA-RZ and HN-RZ) also displayed similar T-RFLP profiles, in which 16 common T-RFs were detected. The T-RFLP profiles for both of these samples were strongly dominated by a 389-bp *Hha*I T-RF and a 142-bp *Msp*I T-RF, which resulted in significantly lower Shannon's diversity and Shannon's evenness indices as well as a higher Berger-Parker's index, compared to the rhizome T-RFLP profiles from other sites (p<0.05) (Fig. 2, Table 2). The T-RFLP profiles of the rhizomes from these two sites also showed relatively high similarities regarding their Bray-Curtis indices (0.77 and 0.78 for the *Hha*I and *Msp*I profiles, respectively). Thus, the geographic location where *A. officinarum* was grown contributed to only limited variation in the fungal endophyte communities of the host plant.

**Table 2:** T-RFLP profile-based diversity estimates for the rhizomes of *Alpinia officinarum* Hance plants from various growth sites.

	Shannon diversity index (*Hha*I)	Shannon diversity index (*Msp*I)	Shannon's evenness (*Hha*I)	Shannon's evenness (*Msp*I)	Berger-Parker's index (*Hha*I)	Berger-Parker's index (*Msp*I)
HA-RZ	1.64±0.03a	1.67±0.04a	0.48±0.04a	0.67±0.01a	0.53±0.01c	0.42±0.05d
HN-RZ	1.68±0.02a	1.72±0.1a	0.45±0.01a	0.62±0.06a	0.55±0.01c	0.42±0.04d
QJ-RZ	2.23±0.08b	2.11±0.05b	0.62±0.02b	0.64±0.01c	0.30±0.01b	0.30±0.01ab
GZ-RZ	2.24±0.15b	2.07±0.2b	0.64±0.02b	0.65±0.02bc	0.29±0.01b	0.32±0.01b
GY-RZ	2.05±0.05c	1.89±0.03c	0.71±0.02cd	0.70±0.01c	0.25±0.06a	0.30±0.01a
GX-RZ	2.07±0.05c	1.89±0.1de	0.72±0.03c	0.67±0.03c	0.27±0.01a	0.27±0.01a
KM-RZ	1.99±0.06c	1.93±0.11e	0.71±0.01c	0.69±0.02c	0.27±0.01a	0.29±0.01a
FJ-RZ	2.08±0.09c	1.99±0.04de	0.69±0.01cd	0.67±0.05c	0.27±0.01a	0.28±0.09a

The values were calculated separately based on the data from measurements of three independent rhizome samples for each site and are expressed as the mean ± SD. The letters in each lane indicate significant differences (p<0.05).

### Taxonomic affiliation of T-RFs

A total of 226 sequences, recovered from randomly selected clones containing inserts of fungal ITS rDNA from the clone library constructed with pooled DNA extracted from *A. officinarum* rhizomes, were subjected to T-RFLP analysis involving both *Hha*I and *Msp*I mono-digestion. The fragment accumulation curves showed that the numbers of recovered sequences met the requirement for reaching the plateau of T-RFs patterns for both *Hha*I and *Msp*I mono-digestion (data not shown). From the recovered sequences, a total of 22 *Hha*I- and 13 *Msp*I-specific T-RFs were detected, which were further grouped into 25 distinct OTUs, with each OTU defined as a distinct pattern of peaks in the *Hha*I and/or *Msp*I profile (Table 1). Sequencing was conducted on 1–8 sequences from each OTU (all for the OTUs containing ≤2 sequences and approximately 30% of other OTUs). After checking for sequence quality and chimeras, 63 sequences representing 23 distinct OTUs were used for similarity searches using BLAST. Based on taxa of the similaritiest sequences in GenBank BLAST, 39 cloned sequences representing 14 distinct OTUs could be linked to members of 8 fungal genera at the ≥97% identity level: *Pestalotiopsis*, *Penicillium*, *Fusarium*, *Exserohilum*, *Mycoleptodiscus*, *Colletotrichum*, *Meyerozyma*, and *Scopulariopsi*s. At the ≥92% identity level, 2 additional fungal genera can be added to linkage of another 2 OTUs: *Sebacina* to OTU14 (92%), and *Marasmius* to OTU1 (95%). In addition, sequences from another 2 OTUs (OTU3, and OTU13) showing the highest identities to sequence from a Trechisporales clone with 94% identity, and a Sebacinales clone with 99% identity, could be decided to be potential members of Trechisporales or Sebacinales, respectively. All sequences from OTU2, OTU12, OTU20, and OTU24 also shared ≥97% identities with recorded fungal ITS sequences. However, they could only be decided to be members of Basidiomycota as they showed the highest identities to sequences from uncultured Basidiomycota. The details of the sequence analysis are provided in Table 3. And the GenBank accession numbers of the representative sequences are also presented in Table 3. Specifically, *Hha*I-cleaved T-RFs showed relatively high diversity and discrimination in classifying the fungal taxa. Of the 22 T-RFs detected following *Hha*I cleavage, 15 could be affiliated with a single fungal taxon (Table 3).

**Table 3:** Taxonomic affiliation of T-RFs form rDNA ITS sequences of fungal endophytes of *Alpinia officinarum* rhizomes.

OTUs code	*Hha*I-cleaved T-RFs(bp)	*Msp*I-cleaved T-RFs(bp)	GenBank accession numbers of representative sequences	The similaritiest sequences in GenBank and accession numbers	% Identities	% Coverages
1	389	-*	KF718228 - 33	*Marasmius* sp. CBB-361 (AY216476)	95	96
2	85	101	KF718206 - 10	Uncultured Basidiomycota clone unk101 (GU246997)	98–99	99
3	90	97	KF718262 - 67	Uncultured Trechisporales clone LH132 (GQ268679)	94	80
4	101	85	KF718221	Uncultured Basidiomycota (AM113461)	100	100
5	174	142	KF718239 - 45	*Penicillium herquei* strain NRRL 1040 (AF033405)	99	95
6	153	161	KF718222 - 27	*Fusarium oxysporum* isolate 281 (JN232163)	99–100	97
7	170	131	KF718211-12, 14, 16	*Colletotrichum siamense* strain C1316.6 (JX010258)	99–100	97
8	139	527	KF718260	*Fusarium oxysporum* strain TBCui (JN020659)	99	98
9	325	148	KF718246 -53	*Pestalotiopsis* sp. 1 AE-2013 (KF746109)	99	97
10	170	142	KF718217	*Colletotrichum siamense* strain C1316.6 (JX010258)	100	99
11	241	170	KF718209	Uncultured Basidiomycota clone unk101 (GU246997)	99	98
12	317	142	KF718215	*Colletotrichum siamense* strain C1316.6 (JX010258)	99	99
13	540	170	KF718255 - 56	Uncultured Sebacinales clone 2975a (FJ788853)	99	99
14	222	170	KF718257 - 59	*Sebacina vermifera* strain MAFF305835 (DQ983814)	92	100
15	139	161	KF718219	*Exserohilum* sp. CPO 10.011 (JQ388288)	99	96
16	359	126	KF718238	*Mycoleptodiscus indicus* isolate PA2LL5 (JF736515)	99	96
17	161	94	KF718237	*Mycoleptodiscus* sp. Y16 (KC623561)	99	95
18	160	131	KF718213	*Colletotrichum* sp. IP-56 (DQ780418)	99	100
19	148	323	#			
20	382	151	KF718220	Uncultured Basidiomycota clone unk101 (GU246997)	97	97
21	122	142	#			
22	345	126	KF718254	*Scopulariopsis hibernica* (FJ946484)	97	96
23	337	142	KF718234 - 36	*Meyerozyma guilliermondii* strain JY 45 (KM014587)	99	97
24	80	395	KF718205	Uncultured Ascomycota clone 1159 (HM239833)	79	75
25	139	131	KF718218	*Exserohilum* sp. CPO 10.011 (JQ388288)	99	96

* “-” indicates the T-RF not be detected from the sequences. ^#^ indicates invalid sequencing.

### Distribution of individual fungal taxa

**Figure 3:**
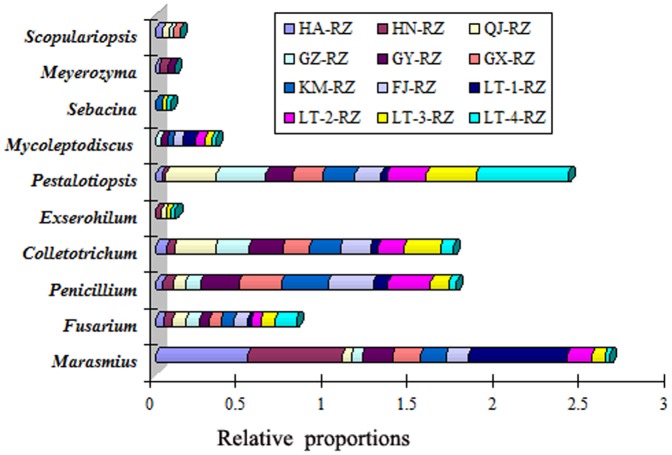
Distribution of individual fungal taxa at the genus level in *Alpinia officinarum* Hance rhizomes. The relative abundance of individual fungal taxa was calculated based on the average values from three independent samples for each year or site.

Based on the relative abundance of each *Hha*I-cleaved T-RF showing linkage to a single fungal taxon, the distribution of individual fungal taxa at the genus level in the rhizomes of *A. officinarum* was analyzed. The most commonly occurring fungal genera in the rhizomes were *Marasmius*, *Fusarium*, *Pestalotiopsis*, *Colletotrichum*, and *Penicillium*, each of which was detected in all of the samples (Fig. 3). The remaining fungal genera were variably represented among the samples. The fungal genera that were predominantly responsible for the difference among the tested samples were *Pestalotiopsis* and *Marasmius*, whose relative proportions in the tested samples varied from 2.2 to 53.6% and 2.7 to 57.8%, respectively (Fig. 3).

### Detection of active chemicals and active chemical-based sample groupings

Two types of active indicator chemicals, total volatile oils and galangin, were examined in each of the examined *A. officinarum* rhizomes (Table 4). Both types of active chemicals were detected in all of the rhizomes, showing varying contents in the range of 5.13±0.47 to 16.89±0.24 mg/g for galangin and 2.98±0.53 to 14.65±0.71 mg/100g for volatile oils. According to the contents of the two types of active chemicals, all of the *A. officinarum* rhizome samples could be divided into four groups. Rhizomes from 4-year-old LT *A. officinarum* (LT-4-RZ) were classified as the high-content group, as they displayed significantly higher active chemical contents than the other rhizomes (the volatile oil and galangin contents were 0.40–3.92 and 0.30–2.29 times higher, respectively, in LT-4-RZ than the other rhizome samples). Rhizome samples GZ-RZ, QJ-RZ, and LT-3-RZ were classified as the sub-high content group, GX-RZ, GY-RZ, FJ-RZ, KM-RZ, and LT-2-RZ as the intermediate content group, and LT-1-RZ, HA-RZ, and HN-RZ as the low content group. The contents of both of tested chemicals showed no significant difference (p≥0.05) among samples from the same group. However, a continuous decrease in high, sub-high, intermediate, and low content groups in a closely linear manner was observed, whether based on volatile oil content or galangin content.

**Table 4:** Detection of active chemicals in *Alpinia officinarum* Hance rhizomes and active chemical-based sample groupings.

	Volatile oil (mg/100g)	Galangin (mg/g)	Groupings
QJ-RZ	10.44±0.75c	12.95±0.19c	Sub-high
HA-RZ	2.98±0.53a	5.13±0.47a	Low
HN-RZ	3.36±0.18a	5.56±1.09a	Low
GZ-RZ	9.96±0.94c	11.66±0.76c	Sub-high
GY-RZ	7.68±0.24b	9.62±0.65b	Intermediate
GX-RZ	7.28±0.50b	9.76±0.46b	Intermediate
KM-RZ	7.92±0.15b	9.27±0.34b	Intermediate
FJ-RZ	7.75±0.27b	8.88±0.23b	Intermediate
LT-4-RZ	14.65±0.71d	16.89±0.24d	High
LT-3-RZ	10.10±0.39c	12.89±0.14c	Sub-high
LT-2-RZ	7.09±0.26b	9.88±0.35b	Intermediate
LT-1-RZ	3.06±0.08a	5.42±0.62a	Low

Values are expressed as the mean ± SD for measurements of three independent rhizome samples for each year or site. The letters in each lane indicate significant differences (p<0.05).

### Non-metric multidimensional scaling (NMDS) analysis across all samples

**Figure 4:**
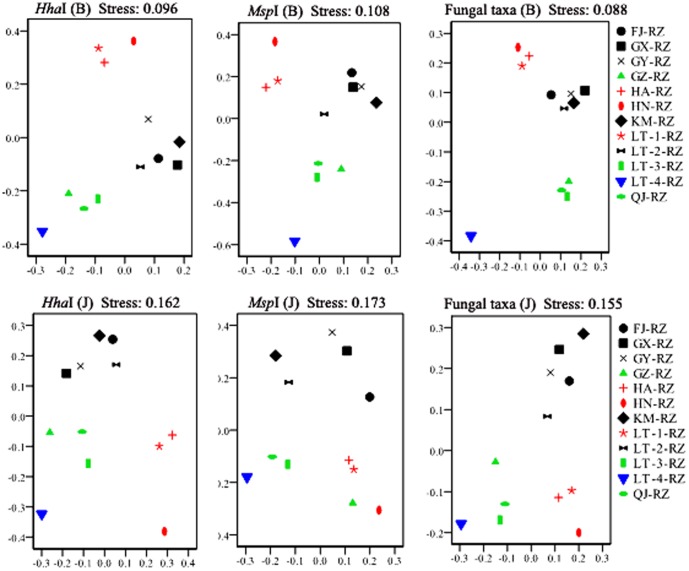
NMDS analysis of fungal endophyte community. Two-dimensional non-metric multidimensional scaling (NMDS) plots using Bray-Curtis similarity index (B) and Jaccard's index (J) showed that the fungal endophyte community assemblages of *Alpinia officinarum* Hance rhizomes could be grouped on the basis of their active chemical contents. The blue, green, black, and red markers correspond to the plots for the *A. officinarum* rhizome samples classified into the high, sub-high, intermediate, and low chemical content groups, respectively.

The Bray-Curtis similarity measure was applied to compare the presence/absence as well as the abundance of T-RFs/individual fungal taxa among samples [37]. The NMDS plots with the Bray-Curtis similarity index clearly divided all of the *A. officinarum* rhizome samples into four groups with low stress values (<0.11) for the three types of datasets representing the fungal endophyte communities detected in the rhizomes (Fig. 4). The LT-4-RZ rhizome sample, which demonstrated a much higher active chemical content than all other samples (Table 4), was well separated from the other rhizomes, forming an individual group. The group memberships in the other three groups were highly consistent with those of the active chemical-based groups (Fig. 4). In particular, the individual fungal taxa-based plots were more tightly clustered within the groups and more discrete between the groups than those resulting from the two other types of data (Fig. 4), whose Bray-Curtis similarities from group membership to group membership were calculated to be >0.85 within the active chemical-based groups, and <0.65 between the active chemical-based groups, respectively. In addition, the separation between the plots from the high-chemicals group (LT-4-RZ) and low-chemicals group (HA-RZ, LT-1-RZ, and HN-RZ) were greater than between the other groups (Fig. 4). Thus, a strong correlation was observed among the active chemical contents and the fungal endophyte communities in the samples.

Jaccard's index only compares the presence or absence of T-RFs/taxa among samples [37]. When Jaccard's index was applied, the NMDS plots from the samples classified as belonging to the same active chemical groups were also similar in most cases (Fig. 4). However, in the Jaccard's index-based NMDS analysis, the plots from several samples classified into the same active chemical groups, but showing highly disparate geographical locations, such as HA-RZ and HN-RZ as well as GX-RZ, GY-RZ, FJ-RZ, and KM-RZ, were more discrete than in the Bray-Curtis similarity-based NMDS analysis (Fig. 4). This result indicated that rhizome samples from different geographic locations could host distinct fungal taxa. However, the fungal taxa that differed among locations did not constitute the dominant taxa within communities of the endophytic fungi from the rhizomes.

## Discussion

Previous studies have demonstrated that T-RFLP analysis of microbial rDNA, which yields high-quality fingerprints consisting of fragments with precise sizes, is a highly reproducible and robust method for studying microbial communities [32], [33], [34]. In this study, the T-RFLP method was used to study the diversity and variation of fungal endophyte communities within *A. officinarum* rhizomes. Although *Msp*I cleavage revealed lower diversity as measured by the number of total T-RFs than *Hha*I cleavage, the differences and similarities in the *Hha*I and *Msp*I T-RFLP profiles among the analyzed samples were generally consistent in most cases. In addition, repeating the complete procedure, from DNA extraction to the final T-RFLP scanning step, showed that the T-RFLP profiles obtained from the single rhizome of a plant were indistinguishable (data not shown). Our results confirmed that T-RFLP analysis is capable of reliably recovering template abundance and overall community structure within natural microbial communities.

The present study was the first to address the fungal endophytes of *A. officinarum*, a medicinal plant with a long history of use. To the best of our knowledge, it is also the first analysis of the endophyte community in plant rhizome tissue using a culture-independent method. Our T-RFLP analyses targeted amplified fungal rDNA ITS sequences from *A. officinarum* rhizomes, yielding a total of 46 distinct T-RFs. At the order or genus level, the T-RFs were associated with 12 distinct fungal taxa. The identified T-RFs only represented ca. 80% of the total T-RFs detected in the ITS clone library, as some T-RFs could only be affiliated with unculturable Ascomycota or Basidiomycota. Thus, it seems likely that the actual number of the fungal taxa present was higher than that detected in the present study. In comparison with the abundance of data on endophytes in non-storage tissues, such as in leaves [8], [10], [11], [14], roots [11], [12], [28], [37], [38], and stems [10], [11], [16], [17], few reports are focused on diversity of rhizome endophytes. Our present results show that a broad spectrum of fungal endophytes can colonize internal tissue of rhizomes, comparable to what has been found in the roots of other plants. For example, using molecular methodology, Kwaśna et al. (2008) recovered a total 20 fungal taxa from five forest tree roots, recording a mean of 11.7±3.4 fungal taxa [37]. The calculated Shannon-Wiener diversity index for the present samples varied from 1.49±0.03 to 2.24±0.15, similar to previous results found in roots, which varied from 0.39 to 2.25 [37].

It is notable that a high proportion (80/226) of the rDNA ITS clone sequences recovered in the present study showed relationship with Basidiomycota fungi. Many cultivation-based studies have isolated Basidiomycota from plant tissues at very low frequencies (<5%) [1], [10], [16]. The possibility of surface DNA contamination was minimized in the present results because the outer layers of the sampled rhizomes were entirely removed before DNA extraction. Thus, the high proportion of Basidiomycota detected here may reflect our use of a culture-independent method, because the direct PCR method has been demonstrated to be capable of effectively recognizing endophytic fungi belonging to the phylum Basidiomycota [37], [38]. An alternative explanation is related to the plant species and tissue type sampled, as several previous studies have demonstrated that Basidiomycota are the large group of fungal endophytes in certain plant species. For example, Rivera-Orduña et al. reported that 22.8% of the fungal isolates from *Taxus globosa* belonged to the Basidiomycota [39], and Liu et al. reported that 36.1% of the fungal isolates from *Bletilla ochracea* (Orchidaceae) belonged to the *Sebacina* sp. within Basidiomycota [40].

Secondary plant metabolites are among the most direct stressors encountered by endophytes and would therefore be expected to significantly alter the endophyte community assemblages within plants [41]. However, only a few previous studies have addressed the effect of the variation in secondary metabolite production on endophyte colonization [42], [43]. Whether host secondary metabolites represent a general mechanism that influences endophyte communities remains unknown. In this study, two types of active indicator chemicals responsible for the pharmacological effects of the medicinal plants studied, total volatile oils and galangin, were examined in each of the *A. officinarum* rhizomes. NMDS analysis across all rhizome samples showed that the fungal endophyte community assemblages of the *A. officinarum* rhizomes could be grouped on the basis of the tested chemical contents (Fig. 4). This finding indicated that the active chemical contents of *A. officinarum* rhizomes is an important factor shaping the fungal endophyte communities of the rhizome tissue. There are three possible mechanisms by which the tested active chemicals might influence the fungal endophyte communities of the host plants. The first such mechanism is direct antifungal action of the active chemicals. The chemicals tested herein have proved capable of inhibiting the growth of diverse microbes, including bacteria, yeasts, and filamentous fungi [24], [44], [45], [46]. The ability to tolerate or resist antifungal chemicals may vary among the endophytic fungal species within a plant, which could influence the fungal communities residing in tissues/organs with different chemical contents. Another possible mechanism is the regulation of reactive oxygen species (ROS) levels by the active chemicals in the host's internal environment. Many past studies have demonstrated that galangin possesses strong antioxidant activity, both *in vitro* and *in vivo*, as well as free radical-scavenging activity [24], [25], [47], [48]. It has been noted that the ROS balance could be critically important for establishing and maintaining the symbiosis of a microbial community [49]. Thus, differences in galangin accumulation might predictably modify the fungal communities within the associated tissues. Finally, the active components influencing the fungal endophyte communities may be affected by interactions among the fungal endophytes. Detoxification of the antifungal compounds produced by the host plant carried out by certain endophytic fungal species can facilitate their own survival and growth as well as that of other endophytic fungal species co-located within these tissues, and thus, could predictably modify the endophyte assemblages [41].

In conclusion, *A. officinarum* rhizomes are colonized by diverse fungal endophyte communities. The diversity of the fungal endophytes found in the *A. officinarum* rhizome appeared to vary with differences in the growth sites and plant age and was closely correlated with the accumulation of active chemicals in the host plant tissue. The variation of the fungal endophyte communities associated with differing growth sites and and plant age is perhaps attributable to differences in the accumulation of active chemicals in the plant tissues.

In the present study, only a culture-independent method was applied and the protocol used in this study was standard when the study was conducted, which may misrepresent particular species due to PCR bias [50]. Again, a reliable identification of fungal taxa by sequence data heavily depends on the availability and quality of reference data from reliably identified strains or specimens. However, only about 14% of the known and less than 1% of the expected fungal diversity were represented by reference sequences in public sequence databases so far and a significant proportion of the published sequences has been shown to be from isolates that were incorrectly named [17], [51]. To give the most comprehensive view of the endophyte community of a plant, future studies will likely require a combination of culture-based method and a more robust designs that can now be accommodated because of increased capacity for sequencing and bioinformatics. Next-generation sequencing technologies, such as metagenomics, which has revealed great diversity within microbial communities [8], [15], [17], [28] and will allow for more economical and comprehensive insights into fungal endophyte communities in the near future, can be explored in future work.

## Supporting Information

S1 Fig **Geographical locations from which** ***Alpinia officinarum*** **rhizomes were collected in present study.** KM: Kunming Botanic Garden, Yunnsn Province, China (25.1433, 102.7417); FJ: Anhai, Fujian Province, China (24.7206, 118.4750); GX: Liang Qing Town, Nanning, Guangxi Province, China (22.5311, 108.3900); GY: Huodao Town in Gaoyao County, Guangdong Province, China (22.8792, 112.3894); GZ: South China Botanical Garden, Guangzhou, China (23.1800, 113.3647); HN: Wanning, Hainan Province, China (18.7350, 110.2328); QJ: Qujie Town in Xuwen County, Guangdong Province, China (20.4528, 110.3619); LT: Longtang Town in Xuwen County, Guangdong Province, China (20.3350, 110.3122); HA: Hai'an Town in Xuwen County, Guangdong Province, China (20.2831, 110.2089).
(TIF)
