# Supplementary figures and images for: Fungal Endophytes of Alpinia officinarum Rhizomes: Insights on Diversity and Variation across Growth Years, Growth Sites, and the Inner Active Chemical Concentration
Source: PLoS One. 2014 Dec 23;9(12):e115289. doi: 10.1371/journal.pone.0115289 (PMC4275216; doi:10.1371/journal.pone.0115289)

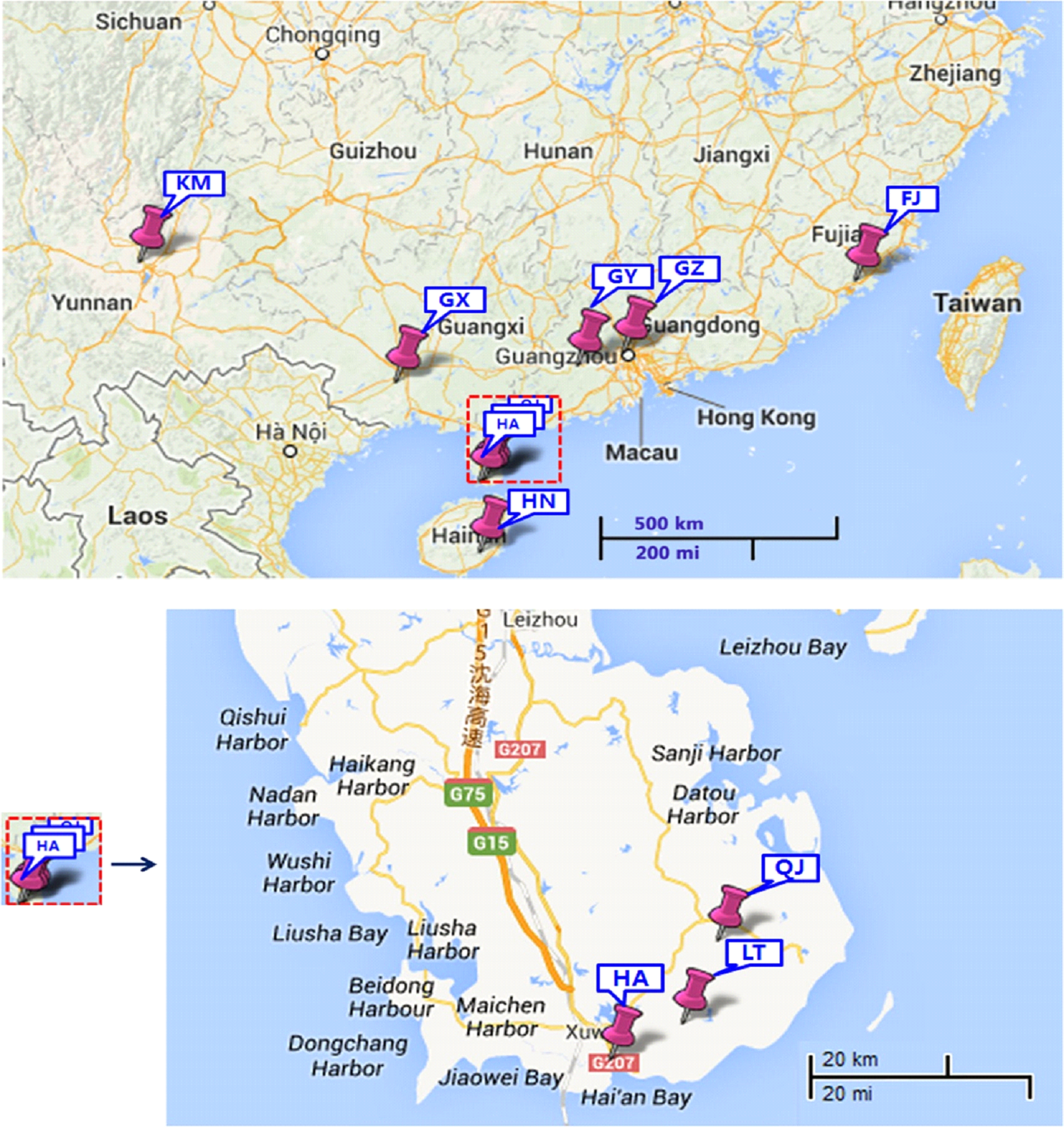

Supplement: S1 Fig — Geographical locations from which Alpinia officinarum rhizomes were collected in present study. KM: Kunming Botanic Garden, Yunnsn Province, China (25.1433, 102.7417); FJ: Anhai, Fujian Province, China (24.7206, 118.4750); GX: Liang Qing Town, Nanning, Guangxi Province, China (22.5311, 108.3900); GY: Huodao Town in Gaoyao County, Guangdong Province, China (22.8792, 112.3894); GZ: South China Botanical Garden, Guangzhou, China (23.1800, 113.3647); HN: Wanning, Hainan Province, China (18.7350, 110.2328); QJ: Qujie Town in Xuwen County, Guangdong Province, China (20.4528, 110.3619); LT: Longtang Town in Xuwen County, Guangdong Province, China (20.3350, 110.3122); HA: Hai'an Town in Xuwen County, Guangdong Province, China (20.2831, 110.2089). (TIF) [file pone.0115289.s001.tif]
